# Career Exploration and Career Decision Self-Efficacy in Northwest Chinese Pre-service Kindergarten Teachers: The Mediating Role of Work Volition and Career Adaptability

**DOI:** 10.3389/fpsyg.2021.729504

**Published:** 2022-01-24

**Authors:** Fangfang Zhao, Ping Li, Siyuan Chen, Yijun Hao, Jinliang Qin

**Affiliations:** ^1^College of Teacher Education, Zhejiang Normal University, Jinhua, China; ^2^Shenzhen Longhua High School, Shenzhen, China; ^3^Hangzhou Preschool Teachers College, Zhejiang Normal University, Hangzhou, China

**Keywords:** career exploration, career decision self-efficacy, work volition, career adaptability, Chinese pre-service kindergarten teachers

## Abstract

Studies have documented that career exploration is significantly associated with CDSE, but how this association occurred is not clear yet. This study committed to clarifying the mechanism underlying the relationship between career exploration and CDSE by investigating the mediation effect of work volition and career adaptability among 586 pre-service kindergarten teachers. The participants are recruited from Ningxia Hui Autonomous Region in northwest China, covering Han, Hui, and other minorities. They took part in a two-wave (6 months apart) longitudinal survey and reported on their career exploration at T1, work volition, career adaptability, and career decision self-efficacy (CDSE) at T2. Results showed that T1 career exploration is directly related to the T2 CDSE. Further, career exploration contributed to the CDSE through both the separated mediation path and the chained mediation path of T2 work volition and T2 career adaptability. The results suggest that individuals who engage in more career exploration activities are likely to have more confidence in their abilities to make career decisions over time, which was partially and serially explained by individuals’ perception of capacity despite constraints and greater self-regulatory strength. This study is a first attempt to deeply clarify the link between career exploration and CDSE, and the findings shed light on the independent and serial mediating effects of work volition and career adaptability. The implications and limitations are discussed.

## Introduction

The shortage of kindergarten teachers (KG teachers) is quite a big challenge to the development of early childhood education (ECE) in China (Chen, 2019). Therefore, as a reserve force, it is very important for pre-service KG teachers (i.e., college students majoring in early ECE) to successfully engage in the profession after graduation. However, the occupation is confronted with a variety of challenges, such as high job stress and low socioeconomic status (Hu, 2020; Li and Li, 2020), and these potential barriers may dampen the confidence in career decision-making (i.e., career decision self-efficacy, CDSE) during the school-to-work transition (Kantamneni et al., 2018; Brown and Lent, 2019; Ulas and Yildirim, 2019). CDSE refers to individual beliefs in the ability to engage tasks associated with the career decision process (Taylor and Betz, 1983; Betz and Luzzo, 1996). Lack of confidence in making the right decisions may lead to unemployment and dissatisfied occupations (Xin et al., 2020). In contrast, high CDSE is significantly related to progress in vocational commitment (Jin et al., 2009) and facilitates smooth career decision-making (Taylor and Betz, 1983; Betz and Luzzo, 1996).

Given the potentially beneficial effects of CDSE to pre-service KG teachers’ career choice, investing in the causes of CDSE may provide key implications for interventions to facilitate successful school-to-work transition. A large number of studies paid attention to the effect of career exploration on CDSE (see Jiang et al., 2019, for a review). Career exploration, defined as “purposive behavior and cognitions that afford access to information on occupations, jobs, organizations that was not previously in the stimulus field” (Stumpf et al., 1983, p. 192), explained the largest variance of CDSE compared to parental support, attachment, and dysfunctional career thoughts (El-Hassan and Ghalayini, 2020). Despite the evidence for the important role of career exploration played in undergraduates’ CDSE, several research gaps await further investigation. First, concerning associations between career exploration and CDSE in the Chinese context, previous studies mainly focused on high school and college students in general (Cheung and Arnold, 2014; Cheung and Jin, 2016; Gu et al., 2020). However, little of the above associations are known between career exploration and CDSE among pre-service KG teachers (most of them are women) who have historically lacked confidence in their ability to make career decisions, especially in Asian countries (Mau, 2000; Quimby and O’Brien, 2004; Shin et al., 2019). Driven by Confucian and Collectivism, Asian students, especially women, tend to choose a job conformed to the expectations of family and society, rely less on individual abilities and self-assertion (Mau, 2000), and are still surrounded by the traditional gender division of labor, women who remain to be the mainstay of domestic work and child rearing. The lower social status forced them to remove some career choices that are incompatible with familial and societal expectations (Shin et al., 2019), have less autonomy in decision-making, experience harsher self-criticism than male students (Betz, 1994; Mau, 2000), and contribute to inadequate confidence in career decision-making. Therefore, it is vital to examine the role of career exploration in the development of CDSE in this group. Second, the intervening mechanisms underlying the link between career exploration and CDSE are not clear yet. It is of great significance to investigate the underlying mechanism in this association from a theoretical and practical perspective.

To address the aforementioned gaps, the current study conducts a two-wave survey to examine the association between career exploration and CDSE, testing work volition and career adaptability as mediators in a sample of Chinese pre-service KG teachers. As a first attempt to clarify the mechanism underlying the relationship between career exploration and CDSE, this study will deepen our understanding of how career exploration is linked to CDSE and will inform intervention programs in practice to enhance pre-service KG teachers’ CDSE. Furthermore, the study will also benefit future career development research and intervention for multiple populations by providing new insights and implications.

## Pre-Service Kindergarten Teachers in China

Over the past decade, ECE development entered a new era and developed rapidly in China (Qi and Melhuish, 2017). The Chinese government has published a large number of policy documents to promote the quality of ECE (State Council of the People’s Republic of China, 2010, 2018). As a key factor affecting the quality of ECE, teachers’ training is also an important policy focus of the Chinese government (Ministry of Education of the People’s Republic of China, 2010). With governmental support, in China, the number of specialized ECE schools and pre-service KG teachers (i.e., college students majoring in preschool education) has nearly doubled in the last 10 years (National Bureau of Statistics of China, 2018). The Chinese government attached great importance to the training and employment of KG teachers (e.g., Ministry of Education of the People’s Republic of China, 2018). Thus, the career development of pre-service KG teachers has attracted much attention over the past few years in China (Wu, 2011). Chinese government enacted relevant policies to improve pre-service KG teachers’ training system and optimize employment conditions to guide them into this profession (Ministry of Education of the People’s Republic of China, 2018). Researchers and professionals also put substantial effort into the improvement of pre-service KG teachers’ career identity and employability (e.g., Chen et al., 2008; Zhao, 2008).

In spite of these outstanding achievements, the career development of pre-service KG teachers is unpredictable. There is a high demand for KG teachers on the job market, yet the recognition of KG teachers as a professional career has not been established in the wide community (Jiang, 2005; Wu, 2011; Boyd, 2013; Taleb, 2013). In China, pre-service KG teachers are still confronted with considerable career barriers when making a career decision, such as unsatisfactory salary level, low social status, little recognition of professional status, ambiguous professional identity, limited employment opportunities, and uncertain long-term career development path (Hu, 2020; Li and Li, 2020), and these may dampen their confidence in career selection and ultimately influence their career options (Kantamneni et al., 2018; Brown and Lent, 2019; Ulas and Yildirim, 2019). As a result, the shortage of KG teachers is still a critical problem of ECE in China (Chen, 2019).

## Career Exploration and Career Decision Self-Efficacy

Career exploration is a process whereby individuals engage in an exploration of the self and the environment related to career development (Stumpf et al., 1983). As a foregoing phase of the career decision-making process, career exploration includes gathering information on vocation from the external environment and exploring personal interests, goals, values, and personality traits that link to career development (Zikic and Klehe, 2006). It is considered a primary developmental task in early adulthood (Porfeli et al., 2013) as exploration activities prompt individuals to identify personal interests, goals, potential professions to pursue (Germeijs and Verschueren, 2006), and eventually make career decisions deliberately.

According to career decision and development theories, career exploration is a driver of the career decision-making process, the more individuals explore personal interests, goals, and external information of the world of work, the better confident they will be on making career decisions that are consistent with self-appraisal and career knowledge (Super, 1957; Tiedeman and O’Hara, 1963; Lent and Hackett, 1987; Blustein et al., 1994; Holland, 1997). A large number of empirical studies also showed that career exploration is strongly associated with CDSE (Blustein, 1989; Bartley and Robitschek, 2000; Creed et al., 2007; Rogers and Creed, 2011; Chiesa et al., 2016), to be noticed, career exploration on working environment linked with CDSE after 3–4 months (Cheung and Arnold, 2014), and for students participated in the career exploration intervention course, the confidence in making effective career decisions has increased compared to pre-course and post-course in quasi-experimental research (Cheung and Jin, 2016; Mallinson and Burns, 2019; Gu et al., 2020). A recent study further found that career exploration is the most essential activity that explains adolescents’ confidence in making career decisions (El-Hassan and Ghalayini, 2020). Hence, it has been established that career exploration plays an important role in helping individuals to facilitate their process of career decision-making, enhancing their confidence in job selection, and making appropriate decisions (Zikic and Hall, 2009; Jiang et al., 2019).

## The Underlying Mechanisms Between Career Exploration and Career Decision Self-Efficacy

As an inclusive career theory, Psychology of Working Theory (PWT) postulated that individuals confront many barriers in the process of career exploration and career decision-making, which would demolish them to secure decent work (Blustein, 2008; Duffy et al., 2016). In this process, two psychological factors were proposed as the mediators of the association between contextual factors and vocational outcomes (Duffy et al., 2016), one is conceptualized as work volition, with another known as career adaptability. Previous studies have found that these two variables are related (Buyukgoze-Kavas et al., 2015; Duffy et al., 2015) and work volition significantly predicted career adaptability longitudinally (Autin et al., 2017). Inspired by PWT, we believe that in the process of exploring work-related information and making career decisions, individuals would benefit from these personal career-related attributes. Therefore, in this study, we propose work volition and career adaptability would, respectively, and sequentially mediate the association between career exploration and CDSE.

### Work Volition as a Mediator

As a centerpiece of PWF, work volition is defined as individuals’ perceived freedom to make vocational choices despite constraints (Duffy et al., 2012a,b). For pre-service KG teachers, the perceived ability to overcome barriers and make desirable career choices may play a more important role in their process of career development, considering faced barriers that are difficult to resolve (England et al., 2020), such as unsatisfactory salary level, low social status, little recognition of preschool teachers’ professional status, ambiguous professional identity, limited employment opportunities, and uncertain long-term career development path (Papanastasiou and Zembylas, 2005; Yesil Dagli, 2012; Hu, 2020; Kim et al., 2020; Li and Li, 2020). Therefore, an attitudinal construct is further needed (i.e., work volition) to overcome obstacles. Hence, it is of greater significance to integrate work volition into the career development process of pre-service KG teachers.

Nevertheless, there is no empirical research on the association between career exploration and work volition to date, and some clues can be drawn from other analogous researches. It has been revealed that personal resources, including personal characteristics on psychology capital and career resources availability and access to information that enable individuals to have comprehensive knowledge about career development and situation of the job market (Hobfoll, 1989), are positively related to work volition (Cheung et al., 2018). Career exploration is also an information collection process on personal characteristics and career environment, and it plays an important role in helping individuals to achieve better employment opportunities and deal with the challenges in career transformation (Zikic and Klehe, 2006). It can be deduced that there is a lot of overlapping characteristics between personal resource and career exploration. Therefore, we ratiocinate that career exploration may have a correlation with work volition to a certain extent. Individuals who have a clear awareness about internal attributes and external work environment would have a positive feeling of control and capability that are necessary for career decision-making in the future.

Work volition is related to a variety of vocational outcomes. For example, work volition significantly and positively correlates with CDSE among undergraduates (Duffy et al., 2012a, 2015; Jadidian and Duffy, 2012), and students who feel more volitional in their career selection tend to be more confident in career decision-making. In summary, we propose that pre-service KG teachers’ career exploration is associated with work volition and work volition further relates to CDSE, suggesting that work volition may serve as a mediator in the career exploration–CDSE relationship.

### Career Adaptability as a Mediator

Grounded in career construction theory (CCT), career adaptability is identified as a psychosocial construct that indicates a person’s readiness and resources for coping with current and anticipated occupational development tasks, transitions, and traumas (Savickas, 2013). It includes four adaptability resources: concern, control, curiosity, and confidence (Savickas and Porfeli, 2012). Career adaptability is an essential resource for individual career development, especially when confront with challenges in work (Johnston et al., 2016), and may increase the chances of graduates finding suitable jobs and facilitating the transition from school to work (Koen et al., 2012; Rudolph et al., 2017). It is vital to explore how career adaptability resources may relate to vocational development, especially for those who faced advanced school-to-work transition because a long-term internship is a prerequisite, such as pre-service KG teachers (Wu, 2011).

As a self-regulation resource, career adaptability had a close association with career exploration. The CCT postulated that to develop career adaptive abilities, individuals tend to constantly enhance self-awareness and get familiar with the work environment through personal experiences (Savickas, 2013), empirical research also indicated that undergraduates with a high level of career exploration showed more adaptability for challenges, and specific career exploration behaviors improve the development of career adaptability over time (Guan et al., 2015). Therefore, there may be a sequential chain from career exploration to career adaptability and resulting career outcomes (Rudolph et al., 2017; Johnston, 2018).

In addition, theorized as self-regulatory strength, career adaptability is a malleable resource to improve personal subjective feeling and well-being (Rudolph et al., 2017; Johnston, 2018). Previous studies have noted that career adaptability links with various positive career outcomes, including CDSE. Career adaptability has a positive correlation with CDSE (e.g., Hou et al., 2014; Duffy et al., 2015; Guan et al., 2016), the greater career adaptability a person has for his/her general career development, the more self-efficient the person would be in their career decision-making. Therefore, it is possible that career adaptability may serve as a mediator between career exploration and CDSE.

### Work Volition and Career Adaptability

Not only work volition and career adaptability are being associated with CDSE, but also studies have found that work volition and career adaptability are related to one another (Buyukgoze-Kavas et al., 2015; Duffy et al., 2015). It suggests that work volition and career adaptability may interact and then contribute to CDSE. There are two potential mediating effects that should be considered. Career adaptability increases CDSE through work volition, and work volition influences career adaptability, which in turn enhances CDSE. The latter seems plausible as it was found that undergraduates’ career adaptability mediated the link between work volition and employability (Kwon, 2019). This finding suggests that it is more feasible to treat work volition as a factor for career adaptability than the opposite. This notion is further supported by a longitudinal study undertaken by Autin et al. (2017) who explored the association between work volition and career adaptability over time (i.e., three-wave). The result showed that there was not reciprocal effect as only work volition (Time 1 and Time 2) significantly predicted career adaptability (Time 2 and Time 3). Based on the previous studies, we postulate that career exploration contributes to CDSE *via* the serially mediated effects of work volition and career adaptability.

## The Present Study

**FIGURE 1:**
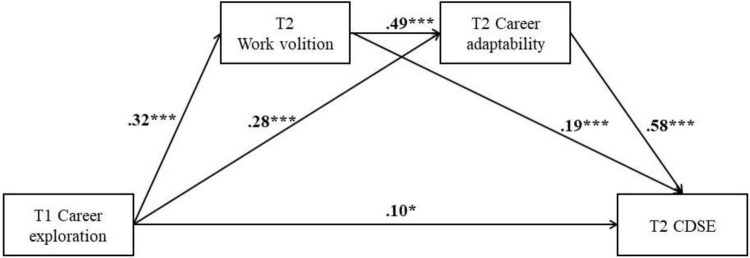
Standardized parameter estimates of the effects of career exploration on CDSE mediated by work volition and career adaptability. *p < 0.05, ***p < 0.001.

The objective of this study is to investigate the nature of the association between career exploration and CDSE, and whether work volition and career adaptability mediate this link in a causally connected manner. We examine this model with a sample of pre-service KG teachers in China over the course of 6 months (i.e., two-wave). We hypothesized three indirect effects (Figure 1): (1) T1 career exploration → T2 work volition → T2 CDSE, (2) T1 career exploration → T2 career adaptability → T2 CDSE, and (3) T1 career exploration → T2 work volition → T2 career adaptability → T2 CDSE.

## Materials and Methods

### Participants

Participants were recruited from one junior college (3-year schooling) in Yinchuan, located in Ningxia Hui Autonomous Region, northwest China. The initial sample at Time 1 (June 2019, second semester of sophomore year) consisted of 586 female pre-service KG teachers (*M*_*age*_ = 21.94, *SD* = 1.23). The participants were from various ethnic backgrounds: 38.8% were Han (the majority ethnic group in China), 54.5% were Hui (one of the minority ethnic groups in China), and 6.7% were other minorities (e.g., Miao, Zang, Dong). The final sample at Time 2 (December 2019, first semester of junior year) comprised 424 female pre-service KG teachers (*M*_*age*_ = 21.77, *SD* = 1.86), yielding a retention rate of 72%, which is considered acceptable for survey data (Taris, 2000). In this longitudinal sample, 58.5% were Han, 41.3% were Hui, and 0.2% were other minorities (e.g., Miao, Zang, Dong).

### Measures

#### Career Exploration

The Career Exploration Survey (CES; Stumpf et al., 1983) was used to measure career exploration activities in the last 3 months. The CES consists of four subdimensions: self-exploration (five items; e.g., “I’ve been focused my thoughts on me as a person”), environment exploration (six items; e.g., “I’ve obtained information on specific jobs or companies”), intended-systematic exploration (three items, e.g., “I sought opportunities to demonstrate skills”), and amount of information (three items, e.g., “how much information do you have on what one does in the career area(s) you interest in”). Students rated the items on a 5-point Likert-type scale (1 = a little, 5 = a great deal). The empirical studies suggested that CES is a valid research tool for assessing career exploration in China (e.g., internal consistency reliability = 0.92 in Guan et al., 2015). Cronbach’s alpha in this study was 0.93.

#### Career Adaptability

We used a 12-item Career Adapt-Abilities Scale–Short Form (CAAS-SF; Maggiori et al., 2017) to measure career adaptability. There are four subscales that form the CAAS-SF, respectively, a concern (three items, e.g., “Preparing for the future”), control (three items, e.g., “Counting on myself”), curiosity (three items, e.g., “Observing different ways of doing things”), and confidence (three items, e.g., “Learning new skills”). All items were scored on a 5-point Likert-type scale (1 = not strong, 5 = strongest). The CAAS-SF has been translated into Chinese and is widely used, both the subscales and the full scale showed good to excellent internal consistency (ranging from 0.62 for concern to 0.74 for confidence, the adaptability total score was 0.88) (Yu et al., 2020). Cronbach’s alpha in this study was 0.92.

#### Work Volition

The Work Volition Scale-Student Version (WVS-SV; Duffy et al., 2012a) was used to measure work volition. It was comprised of a volition subscale (seven items) and a constraints subscale (nine items). We used only the volition subscale (see also Autin et al., 2017). Sample items include, “I will be able to do the kind of work I want to, despite external barriers.” All items were scored on a 7-point Likert-type scale (1 = strongly disagree, 7 = strongly agree). This measure has good psychometric properties in China (e.g., internal consistency reliability = 0.85 in Cheung et al., 2018). Cronbach’s alpha in this study was 0.82.

#### Career Decision Self-Efficacy

The Career Exploration and Decision self-efficacy-Brief decisional Self-Efficacy scale (CEDSE-BD; Lent et al., 2016) was used to measure CDSE. The scale consists of eight items (e.g., “Making a well-informed choice about which career path to pursue”) and was scored on a 10-point Likert-type scale (1 = no confidence at all, 10 = complete confidence). This measure has been revised and demonstrated applicative in China context (e.g., internal consistency reliability = 0.88 in Hampton, 2006; Wang L. et al., 2018; Wang P. et al., 2018). Cronbach’s alpha in this study was 0.94.

#### Covariates

Participants’ ethnic backgrounds (dummy coded as 0 = Han, 1 = other minorities) and family socioeconomic status (SES) were considered as control variables in all the analyses, since previous studies had showed that these variables are associated with CDSE (e.g., Chung, 2002; Hsieh and Huang, 2014). A composite SES score was created by standardizing parents’ educational (1 = elementary school, 2 = junior high school, 3 = senior high school, including occupational middle school, 4 = up-to-3-year college, 5 = 4-or-more-year university) and occupational levels (1 = no job, 2 = part-time job, 3 = full-time job) (*Z*-score) and adding the standardized scores (Li et al., 2017).

### Procedure

The informed consent was obtained from the participating school management authorities, principals, and students. Data for this study were collected through the WJX online data collection service,^1^ which is a reliable and popular online tool for academic institutes in China. The participants were asked to complete an online survey, which had all questions mandated. This method ensured no missing data in the data collection process. All participants were informed that their participation was voluntary and that information (e.g., name, student number) would be kept confidential. Previous studies on adolescents’ career development adopted a 6-month interval of data collection (e.g., Rogers and Creed, 2011), and we used a 6-month time lag between each wave of data collection. Specifically, we collected the first wave of data when the participants were in the second semester of sophomore year (June 2019) and the second wave of data when the participants were in the first semester of a junior year before they start their internship (December 2019).

### Data Analysis

SPSS 20.0 was used to perform descriptive statistics and correlation analysis. Mplus 7.4 was used for mediation model (Muthén and Muthén, 2012). We selected the chi-square (χ^2^), Tucker–Lewis index (TLI), comparative fit index (CFI), standardized root mean square residual (SRMR), and root mean square error of approximation (RMSEA) as our fit indices. The fir standard for TLI, CFI, RMSEA, and SRMR follows the previous researchers’ recommendations (e.g., TLI ≥ 0.90; CFI ≥ 0.90; RMSEA ≤ 0.10, SRMR ≤ 0.10) (e.g., Weston and Gore, 2006).

## Results

### Descriptive Statistics and Correlations

Prior to testing the mediation model, some initial analyses were conducted. First, skewness and kurtosis of all manifest variables were tested, and all values were less than 1.0. Second, correlations were examined to determine the relation of career exploration to CDSE. In line with our hypotheses (Table 1), T1 career exploration was associated positively with T2 CDSE. T1 career exploration was related positively to T2 work volition and career adaptability, respectively. T2 work volition and career adaptability were correlated positively with T2 CDSE, respectively. Simultaneously, T2 work volition was related positively to T2 career adaptability. In addition, the correlation analysis indicated that ethnic backgrounds and SES did not have any relationships with the study variables, and thus, we decided not to include them in subsequent analyses. These correlations suggest that it is feasible to run the next-step further analyses for mediation effects (Frazier et al., 2004).

**TABLE 1:** Descriptive statistics and correlations.

	*M*	*SD*	Skewness	Kurtosis	1	2	3	4	5
1. Ethnic	−	−	−	−	−				
2. SES	−	−	−	−	0.02	−			
3. T1 career exploration	3.31	0.62	–0.23	0.50	–0.06	0.05	−		
4. T2 work volition	4.54	0.94	0.21	0.88	0.07	0.04	0.34**	−	
5. T2 career adaptability	3.48	0.63	0.06	0.46	0.03	–0.02	0.46**	0.58**	−
6. T2 CDSE	6.80	1.60	–0.05	–0.09	0.07	–0.03	0.42**	0.56**	0.73**

*p < 0.05,**p < 0.01.

### Mediation Model

We first tested the direct path coefficient from career exploration to CDSE without the two mediators. We found that the direct path coefficient is significant (β = 0.41, *p* < 0.001). We then tested a full mediated model with two mediators and direct path from career exploration to CDSE. The result showed that the model fits the data well, χ^2^ (6) = 11.48, TLI = 0.98, CFI = 0.99, RMSEA = 0.039, SRMR = 0.044. As shown in Figure 1, career exploration at T1 is significantly related to the level of work volition (β = 0.32, *p* < 0.001) and career adaptability (β = 0.28, *p* < 0.001) at T2. Work volition at T2 was found to have a positive effect on career adaptability (β = 0.49, *p* < 0.001) at T2. Both work volition and career adaptability at T2 are significantly related to CDSE (β = 0.19, *p* < 0.001; β = 0.58, *p* < 0.001, respectively) at T2. The effect of T1 career exploration on T2 CDSE (β = 0.10, *p* < 0.05) continues to emerge even after controlling the effects of T2 work volition and career adaptability.

### Assessment of Indirect Effects

Bias-corrected bootstrapping CIs were calculated to test the significance of indirect effect (i.e., whether zero is included within the 95% confidence interval). As displayed in Table 2, T1 career exploration exerted its effect on T2 CDSE through the indirect path *via* the simple mediating effect of T2 work volition, T2 career adaptability, and the three-path mediating effect of work volition and career adaptability.

**TABLE 2:** Standardized indirect effects and 95% CI for the mediation model.

Model pathways	Estimated	95% CI	
		Lower	Upper
Career exploration → work volition → CDSE	0.06^a^	0.03	0.10
Career exploration → career adaptability → CDSE	0.16^a^	0.10	0.23
Career exploration → work volition → career adaptability → CDSE	0.09^a^	0.06	0.13

*^a^Bootstrap confidence interval that excludes 0.*

## Discussion

This study examined the association between career exploration and CDSE over time, and the independent and accumulative mediating effects of work volition and career adaptability among 586 Chinese female pre-service KG teachers during their school-to-work transition, a critical stage in their path of career. The findings support the three hypothesized indirect effects: (1) career exploration (T1) → work volition (T2) → CDSE (T2), (2) career exploration (T1) → career adaptability (T2) → CDSE (T2), and (3) career exploration (T1) → work volition (T2) → career adaptability (T2) → CDSE (T2).

This study found that career exploration was associated directly with CDSE longitudinally. In our sample, the practice and attitude of seeking relevant professional information and enhancing self-awareness provided individuals with more reference and support, enhanced their self-confidence, and facilitated their future career decision-making process. This study resonates with the previous research about the link between career exploration and CDSE (Cheung and Arnold, 2014; Cheung and Jin, 2016; Mallinson and Burns, 2019; Gu et al., 2020) and provides new empirical evidence to the notion that this association lasting over time. This finding also echoes the propositions of career decision and development theories which postulate that career exploration plays a vitally driving role in process of career decision. Individuals achieving more information on self and work enable a full-scale picture of career path and contribute to more confidence and assurance in career decision-making (Super, 1957; Tiedeman and O’Hara, 1963; Holland, 1985; Lent and Hackett, 1987; Blustein et al., 1994).

In addition to testing the direct effect, this study is particularly innovative in terms of its focus on the mediating roles of work volition and career adaptability, as a first attempt to illuminate the mechanism that underlies the linkage of career exploration and CDSE. We initiated a hypothesis that work volition and career adaptability would mediate this association over time based on the proposition that engaging in career exploration activities would enhance personal control and adaptability in career development and ultimately prompt self confidence in career decision-making. The results confirm that both work volition and career adaptability play mediating roles in the longitudinal relationship between career exploration and CDSE. These findings fill previous gaps and provide new evidence that career exploration also indirectly promotes CDSE in other ways. It deserves to be noted that after introducing the work volition and career adaptability as mediators, the relation between career exploration and CDSE was still significant and work volition and career adaptability only partially explained the effects.

Work volition at T2 was revealed to be a significant mediator for career exploration at T1 to CDSE at T2. The more the participants explored the information about their career and self, the more sense of control they developed on career selection beyond constraints, and ultimately promoted their confidence in career decision-making over time. Work volition is critical in explaining the association between career exploration and CDSE, and exploration behaviors may promote higher levels of CDSE partially due to the increased perception of volition to choose one’s future career. The result resonates with previous studies on a positive relationship between work volition and CDSE (Jadidian and Duffy, 2012; Duffy et al., 2015). A noteworthy point is that it is the first time we have demonstrated the positive relationship between career exploration and work volition over a period, which in turn affects CDSE. In this sense, work volition is a central factor in one’s career development path, and incorporating the role of work volition into the link between career exploration and CDSE may contribute to new insights into how this relationship works. According to PWF (Blustein, 2008; Blustein et al., 2008), work volition plays an important role in career development journey and outcomes, especially in the manner of career decision-making. People confronted with a variety of barriers that restrict their perception of volition in career decision-making (Blustein, 2008), who freely explore their interests, values, personality, and desired career is on the assumption that everyone has the volition to have a choice, but that is not the truth (Duffy et al., 2014). The deficiency in one’s career development is partially due to a lack of work volition (Duffy et al., 2012b). Its attitudinal construct support work volition is often served as a mediator, bridging the distance between career factors and outcomes (Duffy et al., 2016), as psychological resources play an essential role in processing the influence of external or personal factors. Individuals with increased work volition perceive more capacity to overcome personal or structural barriers and extend the range of potential job opportunities, thereby promoting positive career outcomes (Duffy et al., 2012b).

Career adaptability at T2 was found to play a stronger role in explaining the association between career exploration and CDSE compared to work volition, serving as a significant mediator for career exploration to CDSE. Participants in our sample who engaged in more exploration activities are likely to have more positive beliefs in their abilities to tackle tasks in selecting jobs over time, and linking these behaviors to actual confidence in career decision-making may be partially explained by one’s adaptability resource. The results confirm the significant positive relationship between career exploration and career adaptability longitudinally as the previous study suggested (Guan et al., 2015) and are consistent with the conclusion that career adaptability is significantly associated with CDSE (Duffy et al., 2015). Remarkably, it is also the first time we have revealed the mediating effect of career adaptability in the relationship between career exploration and CDSE. Career adaptability is an increasingly valuable attribute in the ever-evolving world of work (Savickas, 2002), and its mediating role in the relationship between career exploration and CDSE provides new insights into how this relationship occurs and further highlights the significance of self-regulatory strength in the career decision process. Grounded in CCT, career adaptability is theorized as self-regulatory strength (Savickas, 2002). It is an important career-related resource to secure deliberate career decision-making, smooth school-to-work transition, coping with career challenges, and contribute to desirable career outcomes ultimately (Savickas and Porfeli, 2012). Individuals who have sufficient resources of self-regulation, such as regulating individual characteristics or response to the environment, resulting in positive individual resources appraisal (Johnston, 2018) and optimistic attitudes toward one’s future career (Duffy et al., 2016), which may ultimately benefit desired vocational outcomes.

Finally, it is worth mentioning that career exploration is indirectly linked with the CDSE through the chained mediation path of work volition and career adaptability. Engaging in exploration about a career at T1 would likely promote the development of work volition at T2, and work volition contributes to the development of career adaptability at T2 and then facilitates the development of CDSE at T2 ultimately. Specifically, participants in our sample who gained more information about their careers and themselves may have become more volitional in their career choices over time and then developed a stronger self-regulating career advantage, ultimately increasing their confidence in choosing better jobs. These results suggest that not only work volition and career adaptability are building blocks of CDSE, but also the sequential effect explains why career exploration links to CDSE over time. As the first endeavor to integrate work volition with career adaptability as chain mediators, this study shed light on the literature by exploiting more possible pathways in understanding the linkage of career exploration and CDSE, deepens the correlation between work volition and career adaptability, and extends previous theoretical propositions by offering a more comprehensive insight into how these two psychological characteristics integrate to facilitate individuals’ career development. According to the core perspective of PWT (Duffy et al., 2016), people confront with many barriers in career decision-making, especially undergraduates. Embedded in this context, Duffy et al. (2016) proposed that work volition and career adaptability both independently act as mediators linking contextual factors to positive career outcomes. However, Autin et al. (2017) revealed that work volition may influence the development of career adaptability, and there existed predictive effects between them. Our study resonates with this conclusion and found that work volition is through the effect of career adaptability to prompt positive vocational outcomes. It is probably because the perceived freedom in career decision boosts the development of self-regulatory strength and then influences downstream career outcomes (Autin et al., 2017).

In sum, this study resonates with the past findings regarding the relationship between career exploration and CDSE. The critical contribution of this study is providing new insights into the literature that work volition and career adaptability independently and accumulatively mediate the career exploration, CDSE linkage, and career exploration is long-acting driver of CDSE in the population of pre-service KG teachers.

## Implications

This study makes important contributions to the literature in at least two ways. First, the effect of career exploration on CDSE is well documented among general samples in the literature, but less is known among the particular population, especially those who lack confidence in their career decision-making. In this study, we explored the longitudinal effect of career exploration on CDSE in mainland Chinese pre-service KG teachers, which contributes to the knowledge of the CDSE field by adding new evidence to the existing literature. Second, we direct specific attention to the role of pre-service KG teachers’ work volition and career adaptability in the association between career exploration and CDSE, and clarify their mediating mechanisms for the first time, providing a new viewpoint for a comprehensive understanding about the question of why career exploration activities benefit individuals experience more confidence in career decision-making.

In practice, our findings may be instructive for educators and counselors working with students who have insufficient confidence in career decision-making, especially for pre-service KG teachers. Considering the independent and accumulative strength of work volition and career adaptability in the larger structural equation model, these factors should be considered in future career intervention programs. First, both work volition and career adaptability are malleable, and counselors and educators can help pre-service KG teachers to have a deliberate decision by guiding them to adjust their perceptions of abilities despite constraints and explore their psychological self-regulatory resources. Second, career exploration plays an important direct and indirect role in promoting the confidence in career choice, and counselors and educators should encourage pre-service KG teachers to explore more information about self and environment and supply them with more pathways to access information, such as career guidance course, internship, and other activities. These efforts would facilitate pre-service KG teachers’ career development directly and through the indirect effect *via* work volition and career adaptability to promote their confidence in career decision-making.

## Limitations

These results presented above should be reviewed in a consideration of several limitations that will inspire future research. First, our sample only consists of female pre-service KG teachers in China. Future researches are expected to examine these results in more diverse groups from different countries. Second, this study only focused on two mediators (i.e., work volition and career adaptability), these conceptualized as in-person factors, and future studies might focus on the contextual variables, such as social support (Choi et al., 2012; Metheny and Mcwhirter, 2013), prompting the further understanding of the association between career exploration and CDSE by connecting the contextual factors with in-person factors. Third, the data are simply based on undergraduates’ self-reports and could be subjective, and future researches are expected to collect data from different paths. For example, a behavior experiment design recruited teachers, classmates, or parents as assessors to examine the individuals’ career-related variables, decrease the bias of data, and increase the accuracy. Finally, this study only has the tracking data at two time points, and the baseline level of the measured variables at T1 is not strictly controlled, which will affect the rigor of the results to a certain extent. Future studies are expected to use more tracking data at different time points to further verify and expand the findings of our study.

## Conclusion

This study is the first to understand how career exploration is related to CDSE among Chinese pre-service KG teachers. This study has explored the association between career exploration, work volition, career adaptability, and CDSE and found that career exploration relates to CDSE longitudinally, and further, work volition and career adaptability partially and chained mediated the association between career exploration and CDSE. We hope these results cultivate future theoretical research and career intervention on CDSE of college students and help them smoothly complete school to work transition.

## Data Availability Statement

The raw data supporting the conclusions of this article will be made available by the authors, without undue reservation.

## Ethics Statement

The studies involving human participants were reviewed and approved by Ethics Review Form for Studies at the Faculty of Psychology, BNU. The patients/participants provided their written informed consent to participate in this study.

## Author Contributions

FZ, PL, and JQ developed the study concept. FZ and PL implemented the study, collected and analyzed the data, and drafted the manuscript. SC, JQ, and YH revised the manuscript. All authors interpreted the results and approved its final version for submission.

## Conflict of Interest

The authors declare that the research was conducted in the absence of any commercial or financial relationships that could be construed as a potential conflict of interest.

## Publisher’s Note

All claims expressed in this article are solely those of the authors and do not necessarily represent those of their affiliated organizations, or those of the publisher, the editors and the reviewers. Any product that may be evaluated in this article, or claim that may be made by its manufacturer, is not guaranteed or endorsed by the publisher.

## Footnotes

1
www.wjx.cn
